# Contemporary biomimetic and bioactive materials in paediatric dentistry: a narrative review

**DOI:** 10.1007/s44445-026-00206-3

**Published:** 2026-07-01

**Authors:** Melis Ünsal-Çalışkaner, Aydın Emre Lermi

**Affiliations:** 1 Department of Paediatric Dentistry, Faculty of Dentistry, Atlas University, Istanbul, Turkey; 2 Faculty of Dentistry, Atlas University, Istanbul, Turkey

**Keywords:** Biomimetics, Paediatric dentistry, Bioactive materials, Glass ionomer cement, Zirconia crowns, Bioceramic, Giomer, Self-assembling peptide, Adhesive dentistry

## Abstract

Biomimetic dentistry seeks to restore the structure and function of natural tooth tissues using materials that closely approximate their biological counterparts. Despite the growing clinical adoption of materials marketed as “biomimetic” or “bioactive,” a persistent terminological ambiguity between these two terms has complicated evidence synthesis and clinical decision-making, particularly in the paediatric context. This narrative review, in which no systematic risk-of-bias assessment was performed, proposes a conceptual two-tier classification framework for biomimetic dental materials, distinguishing passive biomimetics, which replicate physical tooth tissue properties without inducing a biological response, from bioactive substances, which actively promote healing or remineralisation. Passive biomimetics encompass dental ceramics and resin-based composites. Bioactive substances are further subdivided into ion-releasing materials, bioceramic-based substances, biomolecular/bioinductive systems, and smart adhesive/hybrid systems. Each category is appraised against the FDI 2022 Policy Statement criteria for bioactive restorative materials. The proposed classification has not been prospectively validated and is intended as a conceptual framework to guide clinical reasoning and future research. Evidence quality varies considerably across categories: while materials such as glass ionomer cements and mineral trioxide aggregate are supported by extensive clinical data, emerging approaches including self-assembling peptide P11-4 and bioactive adhesive systems remain primarily supported by in vitro or short-term evidence. Future research should prioritise long-term randomised controlled trials in paediatric populations to validate the clinical relevance of emerging biomimetic strategies.

## Introduction

Biomimetics is the imitation of natural tissues to create synthetic alternatives. In biomimetic restorative dentistry, two principal aims are identified: restoring damaged tissue with a restoration that approximates natural tissues as closely as possible and reinstating the biomechanics of the natural tooth. With the development of biomimetic materials, the “extension for prevention” principle has been replaced by a minimally invasive approach (Singer et al. 2023).

As biomimetic restorations aim to replicate the natural appearance of teeth without compromising functionality, materials that mimic the aesthetics of natural dental tissues, such as ceramics and adhesive restoratives, may also be classified as biomimetic (Zafar et al. 2020). A critical challenge in the current literature, however, is the frequent conflation of two conceptually distinct terms: “biomimetic” and “bioactive” (Alluhaidan et al. 2026). A biomimetic material replicates a structural, optical, or mechanical characteristic of a natural biological tissue; it may or may not elicit a biological response. A bioactive material, by contrast, triggers a measurable, beneficial, and specific biological effect in living tissue through a defined mechanism of action, a distinction formalised by the FDI World Dental Federation in its 2022 Policy Statement on Bioactive Restorative Materials (Schmalz et al. 2023). This terminological ambiguity has led to inconsistencies in material classification across published reviews, complicating evidence synthesis and clinical decision-making (Alluhaidan et al. 2026). It should be acknowledged that the biomimetic terminology itself remains subject to debate, with some authors arguing that the term has become overly broad and insufficiently specific for scientific classification purposes (Alluhaidan et al. 2026; Zafar et al. 2020).

The biological characteristics of primary and young permanent teeth, including thinner enamel and dentine, larger pulp chambers, and open apices, demand materials that are not only structurally compatible but capable of supporting ongoing biological processes such as remineralisation and pulp vitality maintenance (Abozaid et al. 2026; Singer et al. 2023). No published review has proposed a unified, paediatric-oriented classification that simultaneously accommodates passive biomimetic materials and bioactive substances, while critically appraising each category against accepted international definitions. This represents a meaningful gap in the current literature.

The term “passive biomimetics” is proposed here to describe materials that replicate the biomechanical and functional properties of teeth without eliciting a bioactive response, as distinct from bioactive materials under the FDI 2022 definition (Abozaid et al. 2026; Schmalz et al. 2023)The present narrative review therefore aims: (1) to clarify the conceptual distinction between biomimetic and bioactive materials in the context of paediatric dentistry; (2) to propose a clinically applicable two-tier classification framework grounded in mechanism of action and FDI criteria(Schmalz et al. 2023); (3) to evaluate the paediatric evidence base for each material category, including emerging approaches such as self-assembling peptides; and (4) to identify priorities for future clinical investigation.

## Methodology

This narrative review was conducted and reported in accordance with the Scale for the Assessment of Narrative Reviews (SANRA) (Baethge et al. 2019). A structured literature search was conducted across four electronic databases: PubMed/MEDLINE, Scopus, Web of Science, and Google Scholar, between July and September 2025. Search terms included “biomimetic,” “biomimetics,” “bioactive,” “bioactive restorative material,” “pediatric dentistry,” “paediatric dentistry,” “pedodontics,” “primary teeth,” “glass ionomer cement,” “zirconia crown,” “mineral trioxide aggregate,” “Biodentine,” “giomer,” “S-PRG,” “self-assembling peptide,” “P11-4,” “enamel matrix derivative,” “dental adhesive,” “remineralisation,” and “pulp therapy,” applied in various combinations using Boolean operators.

Studies were included if they were peer-reviewed English-language publications addressing biomimetic or bioactive dental materials in the context of paediatric or restorative dentistry. Conference abstracts, non-peer-reviewed publications, studies focused exclusively on adult populations, and materials outside the scope of the proposed classification were excluded. Titles and abstracts were screened independently by both authors, with full texts retrieved for eligible articles. Preference was given to systematic reviews and meta-analyses over narrative reviews and single clinical studies. A total of 43 references were incorporated into the final manuscript.

The proposed two-tier classification framework was developed inductively during the initial literature review phase, drawing on the FDI 2022 Policy Statement definitions and the mechanistic properties of identified material categories. The distinction between passive biomimetics and bioactive substances emerged from the observation that existing classifications did not accommodate materials that replicate tissue properties without eliciting a biological response. Following framework development, the literature search was refined to ensure comprehensive coverage of each identified category. It is acknowledged that the final reference selection reflects the authors’ judgement and that no formal risk-of-bias assessment was conducted.

## Conceptual distinction between biomimetic and bioactive materials

A critical precondition for any classification of dental materials under the biomimetic umbrella is a precise description of the terms “biomimetic” and “bioactive,” which are frequently conflated in both the clinical and scientific literature. A biomimetic material is one that replicates a structural, mechanical, optical, or compositional property of a naturally occurring biological tissue. The replication may be passive, producing no direct biological response, or may coincidentally facilitate biological activity as a secondary consequence of structural similarity. The defining criterion is imitation of a natural biological blueprint (Alluhaidan et al. 2026).

A bioactive material, by contrast, is one that triggers a specific, measurable, and beneficial response in a living tissue through a defined mechanism of action. The FDI 2022 Policy Statement provides the most operationally rigorous international definition: a material may only be termed “bioactive” if it satisfies all five of the following criteria simultaneously: (1) the mechanism of action is clearly defined and described (biological, mixed, or chemical); (2) the bioactive effect is scientifically documented in vitro or in situ, and preferably also in clinical studies; (3) the duration of the effect is stated; (4) there are no significant adverse biological side effects, including the development of antimicrobial resistance; and (5) the primary restorative function of the material, the reconstruction of lost tooth form and function, is not impaired (Abozaid et al. 2026; Schmalz et al. 2023).

Applying these definitions reveals that not all bioactive materials are biomimetic, and not all biomimetic materials are bioactive. Dental ceramics are biomimetic by virtue of their enamel-like optical and mechanical properties, yet they are not bioactive under any accepted definition. Conversely, silver diamine fluoride produces a well-documented bioactive cariostatic effect but does not imitate any natural tissue structure. Materials such as calcium silicate-based cements occupy a conceptually intermediate position: they are bioactive in the strict FDI sense, but their designation as biomimetic requires justification (Abozaid et al. 2026).

MTA and Biodentine are classified as biomimetic on the grounds that they stimulate the formation of a dentine-like mineralised matrix through odontoblast-like cell differentiation at the material-pulp interface, functionally imitating the physiological dentinogenic response. This constitutes process-level biomimesis, satisfying a broader definition that encompasses not only structural but biological imitation of natural tissue behaviour (Acharya et al. 2024; Alshalan et al. 2025; Lu and Zheng 2025).

Self-assembling peptide P11-4 represents the most explicit example of molecular biomimesis in contemporary dentistry: its amino acid sequence (QQRFEWEFEQQ) was rationally designed to replicate the extracellular matrix scaffolding conditions that guide hydroxyapatite nucleation during enamel formation, constituting biomimesis at the nanomolecular level. This classification acknowledges that the boundary between biomimetic and bioactive is not always absolute, and that a material may legitimately satisfy criteria for both designations simultaneously (Alluhaidan et al. 2026; Keeper et al. 2023).

## Passive biomimetics

Passive biomimetics encompass dental ceramics and resin-based composites. Dental ceramics possess properties closely approximating those of enamel and dentin in terms of surface hardness and elastic modulus, while providing excellent aesthetics and low biotoxicity (Zafar et al. 2020). Preformed Zirconia Crowns (PZCs) constitute a viable aesthetic alternative to Preformed Stainless Steel Crowns (SSCs), demonstrating good retention, adequate fracture resistance, no unusual attrition of opposing teeth, and superior gingival health (Alrashdi et al. 2022; Hamrah et al. 2021). However, PZCs require more extensive tooth preparation than SSCs (Clark et al. 2016); consequently, for anxious or uncooperative children, SSCs remain the treatment of choice owing to their considerably shorter fitting time (Hamrah et al. 2021).

For the repair of moderate tooth damage, resin-based composites are recommended on account of their compatibility with the minimally invasive philosophy and their outstanding optical and aesthetic properties (Zafar et al. 2020). For Class I cavities, composite restorations exhibit a low annual failure rate and a lower incidence of secondary caries compared to compomers (Hickel et al. 2005; Soncini et al. 2007). When compared to amalgam, composite survival rates are equivalent over the first 3.4 years (Soncini et al. 2007); however, given that primary teeth are exfoliated within a defined timeframe, this period is clinically relevant for treatment planning. In the long term, amalgam demonstrates superior survival rates over composites (Antony et al. 2008), a comparison that is becoming increasingly less relevant in clinical practice. Under Regulation (EU) 2024/1849, dental amalgam was already prohibited for children under 15 years of age as of 2018, with a full EU-wide ban for all patient groups taking effect on 1 January 2025. The sixth Conference of the Parties to the Minamata Convention on Mercury (COP-6, Geneva, November 2025) subsequently established 2034 as the global phase-out deadline, after which the manufacture, import, and export of dental amalgam will no longer be permitted worldwide. Future paediatric restorative decisions will therefore be made exclusively among mercury-free alternatives, consolidating the clinical imperative to optimise biomimetic composite strategies in high-caries-risk paediatric populations. From a handling perspective, composites are technique-sensitive and require a longer placement time compared to amalgam (Donly and García-Godoy 2015), and are more hydrophobic than compomers (Nicholson 2007).

Composite strip crowns are indicated for the restoration of extensively damaged anterior primary teeth. The crown form is fabricated by polymerising resin-based composite within a celluloid strip crown matrix and therefore qualifies as biomimetic. These restorations offer good aesthetics and superior gingival health compared to SSCs and zirconia crowns, with minimal tooth preparation (Almajed 2024). However, their clinical performance is limited by several failure modes specific to the paediatric cases. Crown fracture and chipping represent the most commonly reported complications, attributable to the inherent brittleness of composite resin and its susceptibility to fracture under masticatory forces, a particularly relevant concern given the functional loading patterns of primary anterior teeth in young children (Almajed 2024; Suresh and Gunasekaran 2024). Debonding at the adhesive interface constitutes a further clinically significant failure mode, arising from inadequate moisture control during placement, insufficient enamel surface area for bonding in severely carious teeth, and the hydrophilic oral environment characteristic of cooperative young patients(Suresh and Gunasekaran 2024). Colour instability and surface degradation over time represent additional long-term limitations that compromise aesthetic outcomes (Almajed 2024). In light of these failure modes, composite strip crowns are best considered a medium-term restorative solution, with clinical success highly dependent on case selection, isolation quality, and operator experience.

Composite resins may contain Bisphenol A (BPA) and its derivatives. This industrial chemical poses a hazard to the endocrine system and has been shown to increase in serum within 24 h of dental treatment, returning to baseline within 14 days (Marzouk et al. 2019). Although urinary BPA levels from dental treatment appear substantially lower than those associated with other exposure sources (Tichy et al. 2025), clinical significance has not been definitively established. Clinicians should therefore exercise informed judgement when selecting composite resin materials (Marzouk et al. 2019; Tichy et al. 2025).

## Bioactive substances

**Fig. 1:**
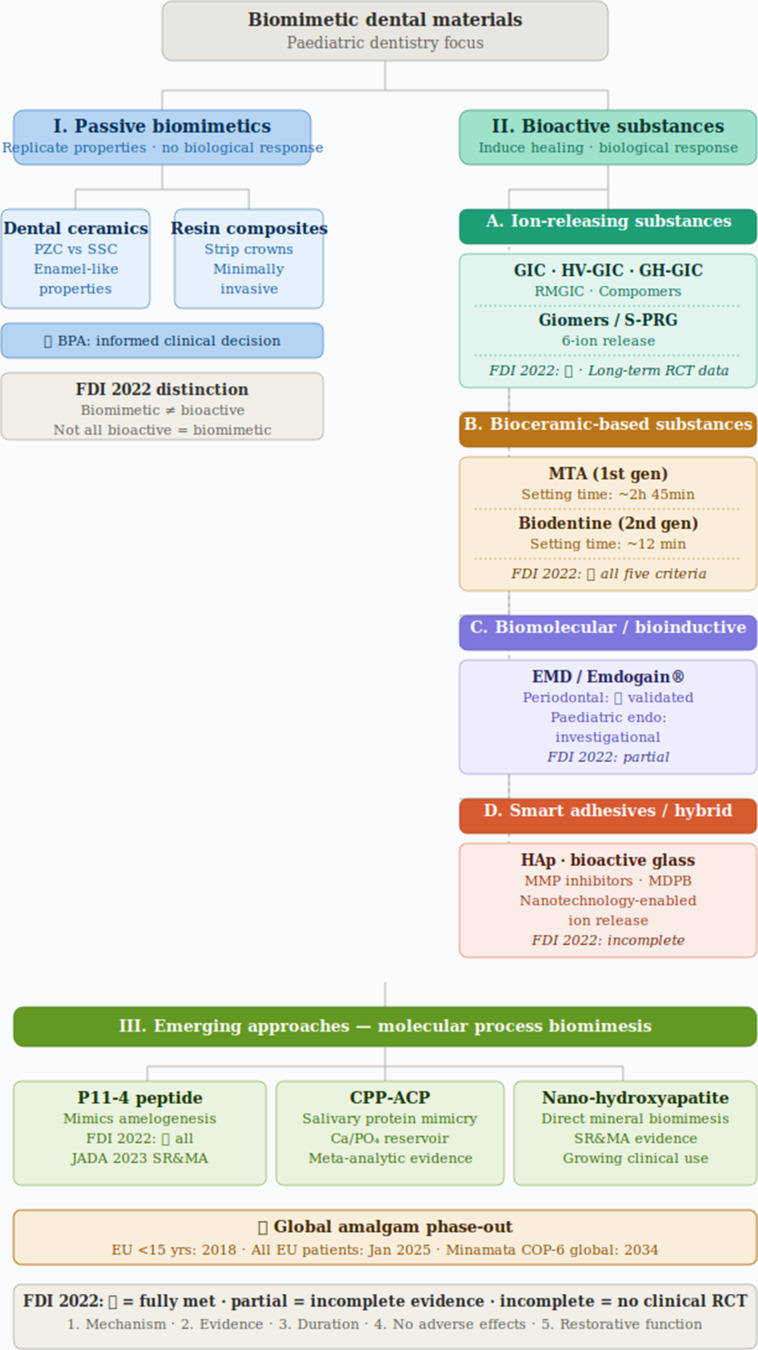
Classification of biomimetic materials in paediatric dentistry

Bioactive substances are distinguished by their ability to promote healing, reinforce, or otherwise trigger a response in living tissues (Abozaid et al. 2026; Schmalz et al. 2023). In this review, materials are similarly grouped by mechanism; however, the classification has been expanded to four subcategories: ion-releasing bioactive substances, bioceramic-based substances, biomolecular/bioinductive systems, and smart adhesives/hybrid systems. The proposed classification framework is illustrated in Fig. 1.

### Ion-releasing substances

#### Glass ionomer cements and resin-modified glass ionomer cements

Glass ionomer cements (GICs) are a family of restorative materials encompassing conventional GICs, resin-modified variants (RMGICs), and polyacid-modified systems (compomers), each sharing the core property of chemical adhesion to tooth structure but differing in composition, mechanical performance, and biocompatibility profile. Conventional GICs are characterised by good biocompatibility and release fluoride ions to support remineralisation and reduce the risk of secondary caries (Abozaid et al. 2026; Ugurlu 2020). They have additionally been shown to release sodium, aluminium, calcium, silicon, and phosphorus ions(Aliberti et al. 2025b; Nicholson et al. 2021). Compared to composite resins, conventional GICs exhibit inferior mechanical properties, limiting their use in load-bearing applications (Zafar et al. 2020).

Among the variations of GICs, high-viscosity glass ionomer cements (HV-GICs) are noteworthy for their increased glass particle content, which enhances translucency and confers superior mechanical and physical properties compared to conventional GICs, enabling their use in load-bearing areas. Next-generation glass hybrid glass ionomer cements (GH-GICs) demonstrate comparable or marginally superior mechanical properties with faster ion release kinetics. These systems incorporate a protective resin coating that plays an important role in surface durability (Arandi 2025).

Resin-Modified Glass Ionomer Cements (RMGICs) were developed to overcome the mechanical limitations of conventional GICs, offering improved strength and reduced working time. Their capacity to absorb water is beneficial where moisture control is challenging; however, biocompatibility is notably lower than conventional GICs due to the release of HEMA (2-hydroxyethyl methacrylate) monomer within the first 24 h, which is cytotoxic to the pulp should it penetrate dentinal tubules. Polyacid-modified GICs (compomers) are less technique-sensitive and exhibit good buffering capacity, but their high-water absorption reduces mechanical strength and results in dimensional expansion over time (Park and Kang 2020).

#### Giomer / S-PRG

Giomer is a collective term for dental materials containing the Surface Pre-Reacted Glass-Ionomer (S-PRG) filler, which confers bioactive properties through a multi-ion release mechanism (Manente et al. 2026). Unlike conventional glass ionomers, the S-PRG filler is pre-reacted prior to incorporation into a resin matrix, yielding a hybrid material that combines the aesthetics and mechanical strength of composite resin with the ion-releasing capacity of glass ionomer systems. As summarised in Table 1, six ions are released by S-PRG, fluoride, sodium, strontium, boron, silicate, and aluminium, each contributing a distinct biological function within the oral environment, collectively promoting remineralisation, antibacterial activity, and acid-buffering effects (Imazato et al. 2023). Giomers have shown high survival rates, minimal postoperative sensitivity, and satisfactory aesthetic performance in primary teeth (Toz-Akalin et al. 2024), and represent a viable alternative to conventional GICs and composite resins in the management of early childhood caries (ECC) (Sesiliana and Riyanti 2021).A recent scoping review confirmed their bioactive effects while noting that long-term clinical evidence remains limited and further randomised controlled trials are needed (Manente et al. 2026).

**Table 1:** Ions released by S-PRG filler and their biological functions in the oral environment

Ion	Biological Function
Fluoride (F⁻)	Remineralisation of enamel and dentine; inhibition of bacterial acid production
Sodium (Na⁺)	Acid-buffering; maintenance of ionic homeostasis at the tooth surface
Strontium (Sr²⁺)	Substitution for calcium in hydroxyapatite; enhanced mineral stability
Boron (B³⁺)	Antibacterial activity; inhibition of biofilm formation
Silicate (SiO₄⁴⁻)	Support of remineralisation; structural contribution to the ion-release matrix
Aluminium (Al³⁺)	Reinforcement of the glass matrix; contribution to ion-release kinetics

S-PRG = surface pre-reacted glass ionomer; HAp = hydroxyapatite

Giomer-based pit and fissure sealants represent an additional area of application. Clinical evidence indicates that prior acid etching significantly improves giomer sealant retention at 12 months, reducing the risk of sealant loss by approximately 69% compared to application without etching (Elmokanen and Gad 2024). Accordingly, the self-etching mode of application, while more convenient, should be used with caution in high-caries-risk children for whom sealant retention is critical.

### Bioceramic-based substances

Bioceramic materials in dentistry refer primarily to calcium silicate-based cements, encompassing Mineral Trioxide Aggregate (MTA) and its derivatives, as well as tricalcium silicate materials such as Biodentine. Their bioactivity is mediated through a well-defined hydration reaction: upon contact with aqueous tissue fluids, calcium silicate (Ca₃SiO₅) undergoes hydration to yield calcium hydroxide (Ca(OH)₂), and a calcium silicate hydrate gel. The resulting alkaline environment and sustained release of calcium and hydroxyl ions create conditions favourable for the precipitation of hydroxyapatite at the material-tissue interface, a process that directly stimulates the differentiation of odontoblast-like cells and the deposition of a mineralised dentine-like matrix (Acharya et al. 2024). This mechanism distinguishes calcium silicate-based bioceramics from ion-releasing materials such as GICs: whereas the latter support remineralisation through fluoride and calcium ion release, bioceramics actively induce hard tissue formation through dentinogenesis and cementogenesis, classifying them as bioinductive agents under the FDI 2022 framework (Acharya et al. 2024; Schmalz et al. 2023).

MTA, the first-generation calcium silicate cement, demonstrates high biocompatibility, excellent sealing capacity, and anti-inflammatory properties in pulp tissue, as well as osteoconductive and cementoinductive characteristics(Acharya et al. 2024). In paediatric endodontics, MTA is one of the most extensively evidenced materials for vital pulp therapy, including direct and indirect pulp capping and pulpotomy in both primary and young permanent teeth. Nevertheless, MTA presents several clinical limitations: a prolonged setting time of up to 2 h and 45 min, challenging handling properties, and a tendency to cause tooth discolouration due to its bismuth oxide radiopacifier, a particularly significant drawback in the aesthetically sensitive anterior region (Alshalan et al. 2025).

Biodentine, a second-generation calcium silicate cement commercially available since 2009, was developed to address the shortcomings of MTA. A clinically significant advantage is its substantially reduced setting time: while conventional MTA formulations require up to 2 h and 45 min to achieve an initial set, Biodentine sets within approximately 12 min, dramatically reducing chair time and the risk of material displacement during restoration placement. Supplied as a capsule system mixed in a triturator, it yields a more consistent and clinically manageable material than hand-mixed MTA. Biodentine promotes the formation of a dentine-like matrix by odontoblast-like cells and has demonstrated an absence of cytotoxicity and genotoxicity (Acharya et al. 2024). A recent systematic review and meta-analysis reported that Biodentine achieved clinical and radiographic success rates comparable to other bioceramic materials in vital pulp therapy of young permanent teeth, while exhibiting a significantly lower rate of tooth discolouration compared to MTA, making it particularly suitable for anterior teeth in children (Lu and Zheng 2025). More recently, premixed bioceramic materials such as NeoPutty MTA (NuSmile) have further improved clinical handling through their non-tacky consistency and resistance to washout (Acharya et al. 2024).

A meta-analysis of first- and second-generation bioceramic materials used in primary dentition pulpotomies confirmed high success rates for both generations, with second-generation materials offering improved aesthetics and handling without compromising clinical efficacy (Albernaz Neves et al. 2025). Calcium silicate cements also remain the preferred materials for apexogenesis and apexification procedures in immature permanent teeth, supporting continued root development and providing a reliable apical barrier (Gerard and Gaur 2025).

### Biomolecular/bioinductive systems

Biomolecular and bioinductive systems constitute a distinct category within bioactive dentistry, in which the biological response is mediated not by ion release but by signalling proteins and growth factors that directly modulate cellular behaviour (Nizami et al. 2025). The most clinically established example is enamel matrix derivative (EMD), commercially available as Emdogain^®^ (Straumann) (Fan and Wu 2022).

EMD is primarily composed of amelogenins, proteins derived from the developing porcine tooth enamel matrix that play a fundamental role in periodontal tissue formation during root development. Its mechanism of action involves mimicking the biological signals of cementogenesis: EMD promotes the proliferation and differentiation of periodontal ligament cells, cementoblasts, and osteoblasts, while stimulating the secretion of key growth factors including TGF-β1, BMP-2, and VEGF (Fan and Wu 2022). Clinically, EMD has been shown to significantly improve periodontal attachment levels and probing pocket depth in intrabony defects compared to placebo, with one-year outcomes demonstrating a mean attachment gain of approximately 1.1 mm (Mayta-Tovalino et al. 2023).

Within the context of paediatric dentistry and endodontics, the use of EMD remains investigational. In vitro evidence demonstrates that EMD can promote the odontoblastic differentiation of dental pulp stem cells (DPSCs) through activation of MAPK signalling pathways, suggesting a potential role in pulp-dentin regeneration (Zhang et al. 2022). However, in vivo comparative studies have shown that EMD is outperformed by calcium hydroxide in terms of hard tissue bridge formation and by MTA in terms of clinical and radiographic success when used as a direct pulp capping agent (Garrocho-Rangel et al. 2009). Accordingly, the routine use of EMD in paediatric pulp therapy currently lacks sufficient clinical evidence and warrants further investigation through well-designed randomised controlled trials.

### Smart adhesives / hybrid systems

Smart adhesives and hybrid systems represent an emerging frontier in biomimetic dentistry, in which the adhesive layer is transformed from a passive bonding agent into a biologically active component of the restoration. These systems incorporate bioactive fillers — such as calcium phosphate nanoparticles, fluorapatite, bioactive glass, and fluoride-releasing compounds, into conventional adhesive resin matrices, enabling the sustained release of remineralising ions at the tooth-restoration interface (Nizami et al. 2025). A defining feature of contemporary smart adhesives is their integration of nanotechnology: nanoparticle-sized calcium phosphate and hydroxyapatite fillers provide dramatically increased surface area compared to conventional fillers, enhancing ion release kinetics and enabling more effective remineralisation of the demineralised dentin collagen network at the nanoscale (Nizami et al. 2025). Commercially available representatives of this category include Clearfil SE Protect Bond (Kuraray Noritake Dental), which incorporates the antimicrobial monomer MDPB, and FL Bond II (Shofu), which contains S-PRG filler for multi-ion release; both systems have demonstrated sustained antibacterial activity and remineralising potential in vitro (Baraka et al. 2024; Nizami et al. 2025). Long-term randomised clinical trials evaluating the performance of these systems specifically in paediatric patients are still lacking, and the following discussion is largely derived from in vitro investigations.

The primary objective of bioactive dental adhesives is the prevention of secondary caries, which remains the leading cause of restoration failure due to hydrolytic degradation of the adhesive-dentin interface and bacterial infiltration. The release of calcium, phosphate, and fluoride ions by these adhesives promotes biomimetic remineralisation of residual demineralised dentin collagen, thereby preserving the integrity of the hybrid layer over time. Additional functional components, including antimicrobial monomers such as MDPB and matrix metalloproteinase (MMP) inhibitors, further enhance the stability and longevity of the adhesive interface (Nizami et al. 2025).

Bioactive adhesives hold considerable clinical promise where caries susceptibility is elevated and optimal isolation challenging. Evidence suggests that their remineralising properties may reduce secondary caries at restoration margins (Baraka et al. 2024); however, scoping reviews of adhesive strategies in primary and young permanent teeth consistently identify a significant gap in high-quality comparative clinical data, underscoring the need for well-designed randomised controlled trials evaluating contemporary bioactive systems in paediatric populations (Delgado et al. 2021).

## Emerging biomimetic approaches

Among the most conceptually sophisticated biomimetic strategies to enter the clinical landscape is the application of self-assembling peptides, of which P11-4, commercially available as Curodont Repair and Curodont Repair Fluoride Plus (vVARDIS), is the most extensively studied. P11-4 is an 11-amino-acid peptide with the sequence QQRFEWEFEQQ, rationally designed to replicate the molecular scaffolding conditions that govern enamel mineralisation during amelogenesis (Alluhaidan et al. 2026). Upon contact with the acidic microenvironment of an early carious lesion, P11-4 monomers self-assemble into a three-dimensional fibrillar biomatrix within the subsurface lesion body, recreating the organic scaffold upon which calcium and phosphate ions deposit as hydroxyapatite, a process that directly mirrors the extracellular matrix-guided mineralisation of natural enamel formation (Alluhaidan et al. 2026; Keeper et al. 2023).

The clinical evidence base for P11-4 has matured substantially. A systematic review and meta-analysis published in the Journal of the American Dental Association confirmed statistically significant superiority of P11-4 over fluoride varnish alone in lesion regression at three- and six-month follow-up, as measured by quantitative laser fluorescence and ICDAS score changes (Keeper et al. 2023). A randomised controlled trial evaluating P11-4 combined with fluoride varnish on white spot lesions in primary teeth demonstrated significantly enhanced remineralisation at six months, with no adverse events reported (Elaziz et al. 2025). These findings satisfy all five FDI 2022 criteria for bioactive material classification, while simultaneously qualifying P11-4 as biomimetic at the molecular level, a dual designation not achievable by any conventional restorative material.

Casein phosphopeptide-amorphous calcium phosphate (CPP-ACP; GC Tooth Mousse) represents an additional biomimetic remineralising system with substantial paediatric relevance. Its mechanism, maintaining a supersaturated calcium and phosphate reservoir at the tooth surface to support biomimetic apatite crystal growth, closely parallels the function of salivary calcium-binding proteins, constituting a biologically inspired rather than purely synthetic approach (Santhosh et al. 2025). A systematic review and meta-analysis comparing CPP-ACP, nano-hydroxyapatite (nHAp), and bioactive glass against conventional fluoride demonstrated that all three agents produced statistically significant remineralisation of initial enamel lesions, with nHAp emerging as particularly effective for white spot lesion management (Santhosh et al. 2025).

These emerging approaches collectively represent a conceptual transition from material-centred biomimetics toward process-centred biomimetics, which seeks to recreate the biological mechanisms by which tooth tissue forms and maintains itself. Long-term randomised controlled trials in primary dentition remain essential before routine clinical adoption can be recommended.

## Discussion

The two-tier classification proposed in this review, dividing biomimetic dental materials into passive biomimetics and bioactive substances, offers a clinically practical framework reflecting both the mechanism of action and the therapeutic purpose of each material (Table 2; Fig. 1). This framework must be applied with awareness of the FDI 2022 criteria: not all materials discussed herein satisfy the full definition of “bioactive” as currently evidenced, with direct implications for the strength of clinical recommendations (Schmalz et al. 2023).

**Table 2:** Summary of biomimetic dental materials: classification, mechanism of action, paediatric indications, advantages, and limitations

Category / Material	Mechanism	Paediatric Indication(s)	Advantages	Limitations
Passive Biomimetics				
Preformed Zirconia Crowns (PZC)	*Enamel optical & mechanical replication*	Full-coverage restoration; primary molars & anterior teeth; aesthetic demand	Superior aesthetics; good retention; better gingival health than SSC; no opposing tooth attrition	Greater tooth preparation; longer chair time; unsuitable for uncooperative children; higher cost
Resin-Based Composites	*Biomimetic optical & mechanical properties; minimally invasive bonding*	Class I–V restorations; primary and young permanent teeth; anterior aesthetics	Excellent aesthetics; minimally invasive; low secondary caries vs. compomers	Technique-sensitive; BPA content; long-term survival inferior to amalgam; hydrophobic
Composite Strip Crowns	*Composite polymerised in celluloid matrix; full-coverage anterior restoration*	Extensively damaged anterior primary teeth; ECC	Good aesthetics; minimal preparation; superior gingival health vs. SSC & PZC	Crown fracture & chipping; debonding at adhesive interface; colour instability; technique-sensitive
Bioactive Substances — Ion-Releasing				
Conventional GIC	*F*,* Ca*,* Na*,* Al*,* Si*,* P ion release; acid-base setting; chemical adhesion*	ART restorations; Class I–II primary teeth; isolation-compromised cases	Fluoride recharge; chemical bonding; biocompatible; low cost; no strict isolation requirement	Inferior mechanical strength; not ideal for high load-bearing areas
HV-GIC / GH-GIC	*Enhanced ion release; increased glass content (HV); resin-coated matrix (GH)*	Load-bearing Class I–II restorations; ART technique	Improved mechanics vs. conventional GIC; faster ion release; surface resin coat improves durability	Still inferior to composite long-term; GH systems: higher cost
RMGIC	*Dual-cure; water absorption; fluoride release*	Difficult isolation; liner/base; core build-up; cervical restorations	Improved strength over GIC; reduced working time; water absorption aids moisture control	Lower biocompatibility than GIC; HEMA cytotoxic to pulp within first 24 h
Compomer (PA-GIC)	*Minimal acid-base reaction; water absorption; polyacid-modified resin matrix*	Class II restorations; primary teeth; proximal surfaces	Less technique-sensitive; good handling; buffering capacity	Lower mechanical strength; dimensional expansion on water uptake
Giomer / S-PRG	*Six-ion release (F*,* Na*,* Sr*,* B*,* silicate*,* Al) from pre-reacted glass filler in resin matrix*	Class I–V restorations; ECC; pit and fissure sealants; high caries-risk patients	Composite aesthetics + ion-releasing bioactivity; fluoride recharge; antibacterial; acid-buffering; FDI 2022: ✓	Long-term clinical evidence limited; sealant retention lower without prior acid etching; higher cost
Bioactive Substances — Bioceramic-Based				
MTA (1st-gen calcium silicate)	*Ca₃SiO₅ hydration → Ca(OH)₂ → hydroxyapatite precipitation; dentinogenesis & cementogenesis*	Pulpotomy; direct/indirect pulp capping; apexification in young permanent teeth	High biocompatibility; excellent sealing; anti-inflammatory; strong clinical evidence base; FDI 2022: ✓	Setting time up to 2h45min; difficult handling; tooth discolouration (bismuth oxide)
Biodentine (2nd-gen calcium silicate)	*Tricalcium silicate hydration; odontoblast-like cell differentiation; dentine-like matrix formation*	Pulpotomy; direct/indirect pulp capping; anterior primary teeth (preferred)	Setting time ~ 12 min vs. MTA; reduced discolouration; capsule mixing; no cytotoxicity/genotoxicity; FDI 2022: ✓	Less long-term data than MTA; higher cost
Premixed Bioceramics (e.g., NeoPutty MTA)	*Calcium silicate-based; hydrophilic set reaction; ready-to-use formulation*	Pulpotomy; indirect pulp capping; convenience-demanding clinical scenarios	Non-tacky consistency; washout-resistant; single-dose delivery; improved handling	Limited long-term RCT data; evidence base still growing
Bioactive Substances — Biomolecular / Bioinductive Systems				
Enamel Matrix Derivative (EMD / Emdogain^®^)	*Amelogenin-based protein signalling; mimics cementogenesis; stimulates TGF-β1*,* BMP-2*,* VEGF*	Established: periodontal intrabony defects. Investigational: pulp-dentin regeneration via DPSC differentiation	Well-validated in periodontics; ~1.1 mm attachment gain at 1 year; FDI 2022: ✓ (periodontal)	Paediatric endodontic use investigational; inferior to MTA/Ca(OH)₂ in pulp capping in vivo; high cost; porcine origin
Bioactive Substances — Smart Adhesives / Hybrid Systems				
Bioactive Dental Adhesives	*Nanotechnology-enabled Ca*,* PO₄*,* F ion release (HAp*,* bioactive glass*,* fluorapatite nanoparticles); MMP inhibition; MDPB*	Secondary caries prevention; primary and young permanent teeth; high caries-risk children	Biomimetic remineralisation of hybrid layer; MMP inhibition improves bond durability; antimicrobial properties	Paediatric RCT data absent; FDI 2022: incomplete (criterion 5 not yet met); lab-to-clinic translation unvalidated
Emerging Biomimetic Approaches — Molecular Process Biomimesis				
Self-Assembling Peptide P11-4	*Rationally designed peptide (QQRFEWEFEQQ) mimics amelogenesis extracellular matrix scaffold; HAp nucleation*	Non-invasive remineralisation of initial carious lesions; white spot lesion management; primary teeth	Molecular-level biomimesis; FDI 2022: ✓ all five criteria; JADA 2023 SR&MA evidence; dual biomimetic + bioactive	Short-term evidence predominant; long-term primary dentition RCTs lacking; higher cost
CPP-ACP	*Salivary protein mimicry; supersaturated Ca/PO₄ reservoir at tooth surface; biomimetic apatite crystal growth*	Remineralisation of initial enamel lesions; white spot lesion management; caries prevention	Established evidence base; meta-analytic support; biologically inspired mechanism; non-invasive	Less potent than P11-4 in some comparisons; formulation-dependent efficacy
Nano-Hydroxyapatite (nHAp)	*Crystallographically identical substitution for lost hydroxyapatite; direct mineral biomimesis*	Remineralisation of initial enamel lesions; caries prevention; white spot lesion management	Direct structural biomimesis; SR&MA evidence; growing incorporation into toothpastes and varnishes	Passive mechanism only; not bioactive under FDI 2022 definition; variable particle size and formulation

ART = Atraumatic Restorative Treatment; BPA = bisphenol A; CPP-ACP = casein phosphopeptide–amorphous calcium phosphate; DPSC = dental pulp stem cell; ECC = early childhood caries; EMD = enamel matrix derivative; GH-GIC = glass hybrid glass ionomer cement; GIC = glass ionomer cement; HAp = hydroxyapatite; HV-GIC = high-viscosity GIC; MDPB = 12-methacryloyloxydodecylpyridinium bromide; MMP = matrix metalloproteinase; MTA = mineral trioxide aggregate; nHAp = nano-hydroxyapatite; PA-GIC = polyacid-modified GIC (compomer); PZC = preformed zirconia crown; RMGIC = resin-modified GIC; S-PRG = surface pre-reacted glass ionomer; SSC = stainless steel crown

Among passive biomimetics, PZCs represent a significant advancement over SSCs in aesthetics and gingival compatibility, though greater tooth preparation and longer chairside time limit their applicability in anxious or uncooperative children (Alrashdi et al. 2022; Hamrah et al. 2021). The long-term survival comparison of composite resins with amalgam must now be contextualised against the global amalgam phase-out: the EU-wide ban effective January 2025 under Regulation (EU) 2024/1849 and the Minamata COP-6 phase-out by 2034 shift the clinical question from “composite versus amalgam” to “which biomimetic composite strategy best replicates amalgam’s durability under paediatric clinical conditions?”

Within the bioactive category, conventional GICs and GH-GICs satisfy all five FDI 2022 requirements, though the duration of fluoride-releasing effect varies across formulations and is significantly influenced by environmental pH and temperature conditions, requiring product-specific evaluation (Abozaid et al. 2026; Aliberti et al. 2025a; Park and Kang 2020). It is notable that evidence quality differs substantially across material categories: conventional GICs and MTA are supported by decades of randomised clinical trial data and systematic reviews, whereas giomers and emerging adhesive systems rely predominantly on short-term clinical studies or in vitro evidence. Giomers satisfy the criteria more comprehensively given their six-ion release profile; however, long-term clinical evidence of sustained bioactivity in the primary dentition remains limited (Manente et al. 2026). The GH-GIC versus giomer decision hinges on clinical context: GH-GIC is preferred for load-bearing posterior restorations, while giomers are preferable for anterior restorations and sealant applications (Arandi 2025; Imazato et al. 2023).

Bioceramic materials, led by MTA and Biodentine, have fundamentally changed the management of pulpal pathology in paediatric patients. Both satisfy all five FDI 2022 criteria for bioactive restorative materials in the context of vital pulp therapy (Schmalz et al. 2023). The transition from formocresol-based pulpotomy agents to calcium silicate-based materials represents a paradigm shift grounded in superior biocompatibility and tissue-regenerative capacity (Albernaz Neves et al. 2025). In selecting between MTA and Biodentine for a paediatric pulpotomy, the critical decision factor is tooth location: Biodentine’s significantly lower discolouration rate and reduced setting time make it the evidence-based preference for anterior primary teeth, while both materials perform equivalently in posterior pulpotomies (Lu and Zheng 2025).

Biomolecular systems such as EMD, while extensively validated in the periodontal context, remain at a preclinical stage with regard to endodontic and paediatric applications (Fan and Wu 2022). Smart adhesive systems represent a conceptually well-founded response to the persistent problem of secondary caries at restoration margins; however, robust clinical evidence in paediatric populations remains limited and the fifth FDI criterion, preservation of restorative function as demonstrated by clinical data, cannot yet be fully satisfied (Nizami et al. 2025). These systems should therefore be considered investigational in the paediatric context.

Emerging approaches, particularly self-assembling peptide P11-4 and CPP-ACP, represent the vanguard of a conceptual transition from structural to molecular process biomimesis. P11-4’s demonstrated clinical efficacy, combined with its dual qualification as both biomimetic and bioactive under FDI 2022 criteria, positions it as a potentially transformative technology for non-invasive paediatric caries management (Elaziz et al. 2025; Keeper et al. 2023). Current evidence, while promising, is predominantly short-term; long-term randomised controlled trials in primary dentition are urgently needed (Santhosh et al. 2025).

Beyond biological and evidentiary considerations, the practical translation of biomimetic concepts into routine paediatric care is influenced by real-world clinical factors. Cost remains a significant barrier for several material categories, particularly PZCs, Biodentine, and self-assembling peptides, potentially limiting accessibility in resource-constrained settings and public dental health programmes. Handling complexity and moisture sensitivity are especially relevant in the paediatric context, where patient cooperation is frequently limited. Operator experience requirements also vary considerably across categories, with newer biomimetic adhesive systems and peptide-based approaches demanding clinical familiarity not yet widely established. These real-world implementation barriers should be considered alongside evidence quality when selecting biomimetic materials in paediatric practice.

Several limitations must be acknowledged. This narrative review is susceptible to selection bias despite the structured search strategy. The evidence base is profoundly heterogeneous: MTA and GIC are supported by decades of randomised trials, while smart adhesives and P11-4 in primary teeth rest primarily on short-term or in vitro evidence. Long-term clinical data from paediatric populations are lacking for most emerging categories, and the proposed classification framework has not been prospectively validated.

## Conclusion

The two-tier classification introduced in this review, passive biomimetics and bioactive substances, provides a coherent conceptual framework for material selection in paediatric restorative dentistry, grounded in mechanism of action and critically appraised against the FDI 2022 Policy Statement criteria, while acknowledging that the clinical applicability of several emerging categories remains contingent upon stronger long-term paediatric evidence. Material selection should integrate evidence-based performance data, patient-specific clinical factors, and the biological objectives of treatment. The global phase-out of dental amalgam consolidates the imperative to optimise mercury-free biomimetic alternatives in paediatric practice, and future research should prioritise long-term randomised clinical trials with particular attention to smart adhesive systems, giomer-based restorations, self-assembling peptides, and biomolecular agents for pulp-dentin regeneration.

Two developments hold particular promise for the next generation of biomimetic dental materials. Advances in nanotechnology are enabling the engineering of restorative materials at the molecular scale, with nanostructured calcium phosphate, bioactive glass, and peptide-based scaffolds increasingly capable of recreating the hierarchical architecture of natural tooth tissues. Concurrently, artificial intelligence-driven material design is beginning to transform the discovery pipeline, with machine learning models now being applied to predict ion release kinetics, optimise filler compositions, and identify novel bioactive compounds. The integration of these technologies into paediatric dental biomaterials science represents an attainable frontier that may ultimately yield restorations capable not only of replicating but of functionally regenerating natural tooth structure.

## Data Availability

No datasets were generated or analysed during the current study.
